# Relationship between sleep, stress and psychological distress in internally displaced persons depends on the length of displacement

**DOI:** 10.1038/s41598-026-57241-7

**Published:** 2026-06-20

**Authors:** Tamar Basishvili, Marine Eliozishvili, Nani Lortkipanidze, Tengiz Tsuladze, Nikoloz Oniani, Nato Darchia

**Affiliations:** 1 Tengiz Oniani Laboratory of Sleep-Wakefulness Cycle Study, Ilia State University, Tbilisi, Georgia; 2 Department of Neurology, Clinic Curatio, Tbilisi, Georgia

**Keywords:** Internally displaced persons, Psychological distress, Sleep, Stress, Length of displacement, Sleep, Trauma, Post-traumatic stress disorder

## Abstract

In recent years, the number of refugees and internally displaced persons (IDPs) has dramatically grown worldwide. We aimed to examine psychological distress and its association with sleep and stress in two groups of IDPs with different lengths of displacement in comparison with the general population. Forty-five individuals displaced in 2008, 2.5 months before this study (2008 IDPs), 67 respondents displaced in 1993 (1993 IDPs), and 53 individuals from Tbilisi general population were assessed. All study participants completed the Insomnia Severity Index (ISI), Perceived Stress Scale (PSS) and Brief Symptom Inventory (BSI). Sociodemographic information such as age, sex, education level and marital status, was collected. The groups differed significantly on age, education, marital status, ISI, PSS, and Global Severity Index (GSI). The highest mean ISI and PSS scores were found for the 2008 IDPs, while the highest GSI score was observed in 1993 IDPs, although the GSI and PSS scores did not differ between the two groups of IDPs. The proportion of people with a GSI T score ≥ 63 was 47.2% in the general population, 71.6% in the 1993 IDPs, and 64.4% in the 2008 IDPs. The ISI and PSS were significant predictors of the GSI in all groups. The PSS exhibited the highest predictive power for 2008 IDPs (β = 0.429), while the ISI - in 1993 IDPs (β = 0.417). Psychological distress persists over time in IDPs. Although both stress and insomnia are strongly associated with psychological distress, perceived stress appears to have a stronger impact in the early resettlement period, whereas insomnia emerges as a more prominent predictor later, years after displacement. Therefore, sleep health in displaced population, and on a broader scale in individuals subjected to traumatic events, warrant attention in long-term perspective for implementing targeted interventions and effectively addressing well-being of affected individuals.

## Introduction

 In recent years, the number of refugees and internally displaced people worldwide has grown noticeably. According to the United Nations High Commissioner for Refugees Mid-Year Trends report, by the end of June 2025, 117.3 million people worldwide were forcibly displaced^1^. This population, confronted with a wide variety of extremely stressful war-related experiences, represents the most vulnerable group for stress-induced negative health consequences^2,3^. Whether displaced across the borders as refugees or within their home country as internally displaced persons (IDPs), these individuals face a range of risk factors for poor health and diminished quality of life^3^. As a consequence, a high prevalence of mental problems, particularly posttraumatic stress disorder (PTSD), depression, anxiety, sleep disturbances and poor social and economic functioning, has been reported in conflict-affected populations in several studies^4–9^.

High rates of sleep problems are consistently observed among populations subjected to traumatic experiences.^9–13^. According to a systematic review of peer-reviewed studies (2000–2018) conducted by Richter and colleagues, sleep disorder prevalence ranged from 39% to 99% among international migrants, refugees, and internally displaced persons (IDPs)^14^. These sleep disturbances may occur transiently, as a normal reaction to trauma exposure, or may last for a longer time^9,13,14^. Importantly, research has shown that the nature of stressors as well as individual vulnerability to stressful events have a major influence on stress-associated sleep pattern alterations^11,15,16^. In turn, sleep loss and sleep disruption are associated with the development and persistence of mental health problems^10,17,18^. Furthermore, sleep disturbances may be found among trauma survivors in the absence of comorbid mental health conditions^19^.

Building on the evidence that stress is a triggering factor of various psychopathologies^10^, individuals with displaced backgrounds are at higher risk to experience psychological distress, commonly defined as a state of emotional disturbance that varies in severity and substantially impairs everyday functioning^20^. While a number of studies have focused on the effects of traumatic experiences on refugees’ psychological distress^3,21^, IDPs have received substantially less attention. Besides, findings regarding the long-term course of psychological distress are inconsistent. For refugee populations, some studies suggest that a longer duration of displacement has a beneficial effect on refugees’ mental health outcomes^8,22^. Conversely, other studies report that psychological distress remains stable for many years and may even worsen with longer periods of resettlement^23–26^. A longitudinal, 12-month follow-up study on psychosocial stress, sleep, and mental health in Syrian refugees in Australia by Lies et al. (2020) demonstrated that sleep symptoms are indirect mediators of long-term mental health problems in this population^27^. Findings on IDPs are similarly mixed. Some evidence suggests that longer displacement is associated with better mental health^3,20,28^, while other data shows a persistence of mental health disturbances for decades^7^. Notably, a significant increase in mental disorders is highlighted in a 1-year follow-up study of IDPs in central Sudan^29^.

Despite this growing scientific evidence, the relationship between stress, sleep, and psychological well-being in IDPs in the context of the length of displacement remains largely unknown. The situation in Georgia provides an opportunity to address this issue by examining IDP cohorts with different lengths of displacement within the same cultural and national context. Georgia has experienced several armed conflicts in recent decades, resulting in the forced internal displacement of more than 268 416 people^30^. Two main war conflicts with the Russian Federation were in the region of Abkhazia, in 1992–1993, and Samachablo, Shida Kartli (the so-called South Ossetia), in 2008. Georgia represents one of the top 25 IDP-generating countries, with IDPs comprising approximately 6.9% of the national population^31^. This context allows for the examination how time since displacement influences the interaction between stress, sleep and distress level.

Therefore, this study aimed to investigate the relationship between sleep, perceived stress and psychological distress levels in two samples of Georgian IDPs with different lengths of displacement compared to the general population.

## Participants and methods

### Participants

This cross-sectional study was carried out in October 2008, 2.5 months after the war conflict in Samachablo, Shida Kartli. The study included 3 groups of participants. Group one, composed of 1993 IDPs, consisted of 67 subjects (mean age 44.9±1.4 years, 52.2% females) displaced from Abkhazia 15 years and 2 months before this study. They were randomly selected from four Tbilisi IDP collective centers – government-owned buildings where groups of IDPs were housed and have remained accommodated. Group two, 2008 IDPs, was formed by 45 IDP subjects (mean age − 35.4±2.02 years, 62.2% females) displaced from Samachablo 2.5 months before the current study. During the assessment period, the group of 2008 IDPs was housed in public buildings, three former kindergartens, before their relocation to villages, which were specifically constructed for IDPs by the government after the 2008 war conflict. Subjects were randomly selected proportionally to the number of families in each building. The sample of the general population consisted of 53 residents of Tbilisi, the capital of Georgia (mean age − 37.4±1.6 years, 66.0% females). They were randomly recruited from one randomly selected district of Tbilisi.

All subjects were provided with detailed information about the research purposes, methods, and time requirements. Individuals were eligible for the study if they were aged 18 years or older, belonged to the target populations (2008 IDPs, 1993 IDPs, or the Tbilisi general population), and provided written informed consent. Respondents were excluded if they were unable to provide informed consent or to complete the interview and fill out the questionnaires due to intellectual/mental impairment, such as severe cognitive deficits or active psychosis that interfered with comprehension of study materials. The study participants received a monetary compensation (GEL15, equivalent to USD10). The study was approved by the Institutional Review Board and was conducted in accordance with the Helsinki Declaration.

### Measures

#### Interview

Face-to-face interviews of the study participants were conducted by trained researchers with the participation of a physician certified in neurology (T.T.). The interviews covered health status and sociodemographic information such as age, sex, education level (high school, college, or university), and marital status (2 groups: single/divorced/widowed and married/cohabiting, dichotomized based on a cohabiting partner to reflect the availability of immediate social support).

In this study, the following questionnaires were administered:

#### Insomnia severity index (ISI)

The ISI is a 7-item questionnaire widely used to assess the severity of insomnia during the last month^32^. Items are evaluated on a 5-point scale (from 0 = not at all to 4 = extremely). Thus, the total score of the ISI ranges from 0 to 28. Scores are classified into 4 categories: absence of insomnia (0–7), subthreshold (8–14), moderate (15–21) and severe (22–28) insomnia. In the present study, the Cronbach’s α of ISI was 0.87.

#### Perceived stress scale (PSS)

 The PSS is a 14-item self-report scale designed to measure the perception of stress during the last month on a 5-point scale (from 0 = never to 4 = very often) to yield a total score ranging from 0 to 56^33^. A higher total score indicates that the individual appraises life as more unpredictable and stressful. The authors suggested that the PSS may be useful as a measure of chronic stress levels^33^. The Cronbach’s α of the PSS in this study was 0.83.

#### Brief Symptom Inventory (BSI)

The BSI is a 53-item instrument designed to assess psychological distress level on a 0–4 point scale^34^. The BSI consists of nine subscales measuring the primary dimensions of psychopathological symptoms. The Global Severity Index (GSI), the average score of all items, is the most sensitive indicator of general psychological distress. The BSI was found to be a relatively reliable and valid cross-cultural measure of psychological distress^35^. GSI scores were also converted to standardized T scores. For adult non-patients, a GSI score equal to or greater than 63 is considered indicative of a distress level that is noteworthy for medical attention^31^. The GSI raw scores and T scores were used in the present analyses.

The ISI and PSS instruments showed good psychometric properties in the Georgian population, with details provided elsewhere^11^. The BSI was translated into Georgian by a bilingual translator using a validated standard translation protocol, followed by a back-translation to check for accuracy, consistency, and equivalence with the original version. The pretesting of the finalized version was performed to further refine the instrument. The reliability analysis was performed using Cronbach’s alpha. In the present study, the BSI showed a very good level of internal consistency with a Cronbach’s alpha of 0.93.

### Statistical analysis

We generated descriptive statistics for all measures using counts and percentages for categorical variables and means and standard deviations for continuous variables. The normality of the variables was tested by the Shapiro‒Wilk test. Differences in the mean scores were assessed using analysis of variance (ANOVA) for normally distributed variables (ISI and PSS scores). For significant ANOVAs, multiple comparisons were carried out with the use of Tukey *post-hoc* analysis. Because the normality of assumptions was not satisfied, BSI total scores were analyzed by the Kruskal‒Wallis *H* test with Dunn’s *post-hoc* test. For ANOVA analyses we report partial eta squared as a measure of effect size. For Cohen’s d, values of 0.2, 0.5, and 0.8 are interpreted as small, medium, and large effect sizes, respectively; for partial η^2^, the corresponding values are 0.01, 0.06, and 0.14. For Kruskal‒Wallis *H* test we report ordinal eta squared, η^2^_H_, which follows the same thresholds as ANOVA eta squared^36^. To determine factors associated with psychological distress, multiple regression analysis was performed in each group with demographics and the ISI and PSS scores as predictor variables. Due to small sample sizes and the number of predictors, we applied bootstrap resampling procedures (BCa 95% confidence intervals, 1000 resamples) and report bias-corrected and accelerated (BCa) 95% confidence intervals for all regression coefficients. There were no missing data points for any of the variables included in the analysis. The significance level was set at α = 0.05. All analyses were performed using PASW Statistics 21.0 (SPSS, Inc., Chicago).

## Results

### Demographic and health variables

The demographic characteristics of the study participants by group are presented in Table 1. The groups were significantly different on age (*p* < 0.001), marital status (*p* < 0.05) and education (*p* < 0.05). The general population was younger than the 1993 IDPs (*p* < 0.01), as were the 2008 IDPs (*p* < 0.001). There was no difference in the mean age between the general population and the 2008 IDPs.

**Table 1:** Comparison of demographic variables between the general population, 1993 IDPs and 2008 IDPs.

Variables	General Population *n* = 53	1993 IDPs *n* = 67	2008 IDPs *n* = 45	Statistics
Age (mean±SE)	37.4±1.60	44.9±1.40	35.4±2.02	F_(2,162)_ = 9.76, *p* < 0.001
Sex n (%)				
Female	35 (66.0)	35 (52.2)	28 (62.2)	*χ*^*2*^*(2)* = 2.54, *p* = 0.281
Male	18 (34.0)	32 (47.8)	17 (37.8)	
Marital status n (%)				
Single/divorced/widowed	25 (47.2)	17 (25.4)	11 (24.4)	*χ*^*2*^*(2)* = 8.12, *p* < 0.05
Married/cohabiting	28 (52.8)	50 (74.6)	34 (75.6)	
Education n (%)				
High school	4 (7.6)	12 (17.9)	9 (20.0)	*χ*^*2*^*(4)* = 10.19, *p* < 0.05
College	7 (13.2)	19 (28.4)	12 (26.7)	
University	42 (79.2)	36 (53.7)	24 (53.3)	

IDPs -Internally displaced persons.

The highest mean ISI score, 11.8, was observed in 2008 IDPs, followed by 9.3 in 1993 IDPs, and the lowest, 6.2, in the general population. The mean ISI score showed significant group differences (F_(2,162)_ = 13.47, *p* < 0.001, η²_p_ = 0.14). Tukey *post-hoc* analysis revealed that all groups differed significantly from each other. Significant group differences were also found for PSS scores, (F_(2,162)_ = 6.35, *p* < 0.010, η²_p_ = 0.07). Recent IDPs displayed the highest mean score for the PSS of 28.2. Scores for 1993 IDPs and the general population were 26.4 and 23.5, respectively. *Post-hoc* analyses indicated that the general population differed significantly from both 2008 IDPs (*p* < 0.01) and 1993 IDPs (*p* < 0.05), whereas no significant difference was found between the two IDP groups (*p* = 0.34).

Regarding psychological distress, the groups differed significantly on GSI scores (χ^2^(2) = 10.996; *p* < 0.01, η²_H_ = 0.06), with a mean rank score of 65.08 for the general population, 91.69 for 1993 IDPs, and 91.17 for 2008 IDPs. A *post-hoc* test showed that the general population exhibited lower distress levels than did the 1993 IDPs (*p* < 0.01) and 2008 IDPs (*p* < 0.001). However, there was no difference on GSI scores between the two groups of IDPs. The proportion of people with a GSI T score ≥ 63 was substantial in all groups − 47.2% in the general population, 71.6% in 1993 IDPs, and 64.4% in 2008 IDPs.

**Fig. 1:**
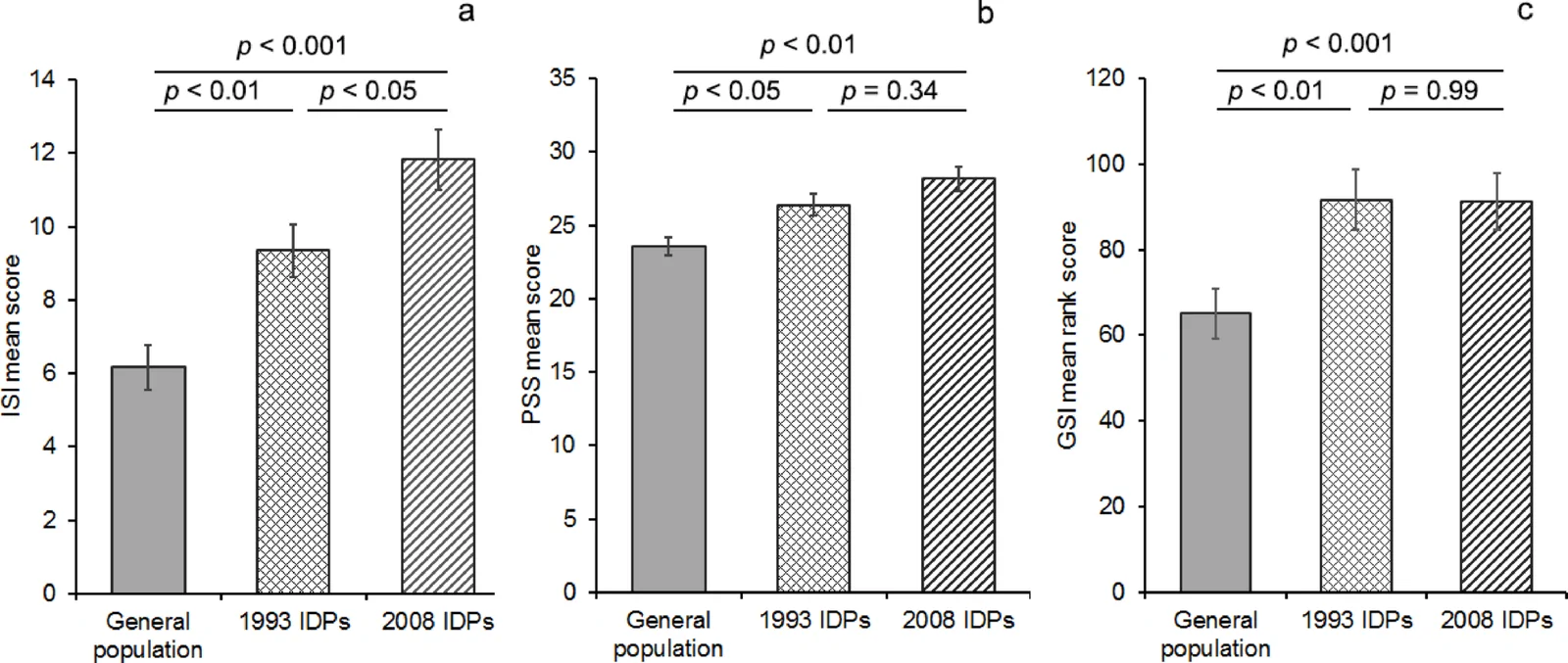
(**a**) Mean values ± SE for Insomnia Severity Index (ISI) scores; (**b**) Mean values ± SE for Perceived Stress Scale (PSS) scores; (**c**) Global Severity Index (GSI) mean rank scores ± SE of ranks. IDPs -Internally displaced persons.

The ISI, PSS mean scores and GSI rank scores according to the groups are presented on Fig. 1.

### Sociodemographic and health predictors of psychological distress 

Multiple linear regression for the GSI, which was performed with demographic variables and the ISI and PSS scores, revealed that the ISI and PSS were both significant predictors of the GSI in all groups. PSS was strongly associated with the psychological status of displaced individuals as well as the general population, with the highest predictive power in 2008 IDPs. The highest predictive value of the ISI for the BSI global severity index was found in IDPs with a much longer period of displacement. In the general population, age and education were also significant predictors. In the displaced population, no other significant predictors were found. The regression model explained 49% of the variance in the level of psychological distress in the general population and 55% and 48% of the variance in the 1993 and 2008 IDPs, respectively. The results of the regression analysis are presented in Table 2.

**Table 2:** Multiple linear regression results for the GSI in the general population, 1993 IDPs and 2008 IDPs.

Predictors	General population		BCa 95% CI	1993 IDPs		BCa 95% CI	2008 IDPs		BCa 95% CI
	β	*p*		β	*p*		β	*p*	
Age	0.246	**0.045**	0.001–0.02	0.109	0.246	-0.004–0.01	0.053	0.682	-0.01–0.02
Sex	− 0.195	0.080	-0.43–0.03	0.002	0.979	-0.23–0.23	− 0.177	0.143	-0.64–0.09
Education	− 0.467	**< 0.001**	-0.59 - -0.19	− 0.080	0.380	-0.21–0.06	0.021	0.861	-0.20–0.24
Marital status	0.016	0.887	-0.21–0.24	0.059	0.514	-0.13–0.33	0.030	0.820	-0.40–0.51
PSS	0.338	**0.003**	0.01–0.06	0.381	**0.001**	0.02–0.05	0.429	**0.008**	0.01–0.09
ISI	0.327	**0.005**	0.01–0.06	0.417	**0.001**	0.03–0.07	0.311	**0.038**	0.002–0.08
**Model significance**	F_(6,46)_- 9.47, *p* < 0.001 adjusted R^2^ **= 0.49**			F_(6,60)_- 14.48, *p* < 0.001 adjusted R^2^ **= 0.55**			F_(6,38)_- 7.68, *p* < 0.001 adjusted R^2^ **= 0.48**		

GSI, Global Severity Index; PSS, Perceived Stress Scale; ISI, Insomnia Severity Index; β, standardized coefficient; BCa 95% CI, Bias-corrected and accelerated (BCa) 95% confidence interval; R^2^, coefficient of determination; IDPs -Internally displaced persons.

## Discussion

Populations exposed to war, armed conflict, and forced displacement are vulnerable to psychological distress and sleep disturbances. This study demonstrates that psychological distress, insomnia severity and perceived stress, all remain elevated in IDPs both shortly after (2008 group) and many years following displacement (1993 group) compared to the general population. Our results show that while the severity of these symptoms is comparable across both IDP groups, the variables that predict distress change over time: perceived stress is the primary predictor in recent IDPs, whereas insomnia is more prominent predictor in long-term IDPs.

Accumulated evidence indicates that the effects of forced displacements are usually associated with significant negative socioeconomic and health impacts on IDPs^3^.^7,31^. These impacts are potentially even stronger in low- and middle-income countries where exposure to socioeconomic adversity and barriers to mental health services are more pronounced^31,37^. One of the main research issues that has been extensively studied in refugee or displaced populations is mental health. The prevalence rates, as well as risk factors associated with mental health in those populations, vary widely, ranging from 8 to 65%, among studies^4,6,7,25,27,38,39^. Most recent evidence shows that sleep disturbances, mental health problems, and specific fears are frequently reported in Ukrainian refugees and IDPs^40,41^.

There is a limited research on the mental health of IDPs in Georgia^5,7,11^. Published studies on the acute and long-term effects of forced displacement on the mental health of Georgian IDPs have demonstrated a high burden of psychiatric symptoms among conflict-affected persons^7^. In this study, we also found that the proportion of people with the GSI score greater than or equal to a T score of 63, which is suggestive of mental health problems, was very high in both IDP groups. Furthermore, there was no significant difference between the two groups of IDPs, despite differences in time since displacement. There are published studies indicating the consistency of mental health problems between long-term and newly resettled refugees^24–26^ or displaced persons^3,7,28^. Poor living conditions, differences in the level of exposure to war-related traumatic events, and a lack of adequate health care may all contribute to the high rate of psychological distress in IDPs with a long period of displacement in the current study. Our results extend findings from other studies by showing high psychological distress in both IDP groups. This is consistent with previous evidence suggesting that psychological distress in forcefully displaced populations may persist long after displacement, supporting the possibility that psychological distress in a conflict-affected population is a chronic rather than an acute condition^24^. On the other hand, we should not exclude the possibility that the war conflict in 2008 became associated with the personal experience of 1993 IDPs and thereby contributed to re-experiencing of their traumatic memories. As such, the war conflict could have had a significant impact on their lives and general well-being. Further research with large sample sizes is required to address the persistence of psychological distress in long-term displaced populations and to elaborate effective health-related intervention programs.

In general, the population with displaced backgrounds shows a greater level of overall psychological distress than does the general population^2,3^. Unexpectedly, the proportion of people from the general population of Tbilisi with a GSI T score of ≥ 63, suggestive of mental health problems, was remarkably high (47.2%). Considering that data collection was conducted 2.5 months after the war conflict that took place in close proximity to Tbilisi (80–100 km), this finding likely reflect elevated psychological symptoms in the general population following the recent conflict.

Two other variables of interest in this study were the PSS and ISI. We found a similar pattern of differences on PSS and ISI scores among groups, with the highest value for both measures in 2008 IDPs and the lowest in the general population. The level of perceived stress did not differ significantly between IDPs with a longer duration of displacement and recently displaced IDPs. These findings suggest that perceived stress is high in displaced population regardless of time since displacement. We may speculate that stress appraisal is related not only to post displacement stressors, which are obviously present in the ID population, but also to the broader consequences of forced displacement, including heightened reactivity to stressful circumstances^21^. As a result, the degree to which they appraise life as stressful is much greater than that of the general population, regardless of the time since displacement.

Perceived stress is related to psychological well-being^42^. It has been suggested that stress appraisal processes, rather than actual stress itself, increase vulnerability to insomnia^11,15^. Previous research conducted in the cohort of 1993 IDPs in Georgia approximately 6–7 months prior to the present study, has reported that insomnia was associated with war-related remembered stress^11^. The stress and insomnia relationship in IDPs becomes more important considering the tight link between stress, sleep disturbances and mental health^10,15,16,25^. The role of sleep in mental health has been demonstrated in a large number of studies^43,44^. Interventions designed to improve sleep may also improve mental health^44^. In the present study, we found that stress and insomnia were both significant predictors of overall psychological distress in the studied groups, with a stronger impact on the displaced population. Other variables, such as age and education, significantly predicted levels of distress only in the general population group. This suggests that socioeconomic benefits typically associated with education, such as higher job positions and better living standards, are less prominent in displaced populations.

Although mean levels of PSS and GSI were similar across both IDP groups, regression analysis revealed a divergence in the predictors of distress. For 2008 IDPs, PSS exhibited the highest predictive value for distress, while ISI emerged as the strongest predictor for 1993 IDPs. These findings provide evidence that both stress and insomnia are associated with psychological distress, with the association of stress being most pronounced in the early resettlement period and the association of sleep becoming more prominent years after displacement. The fact that sleep became relatively more salient in predicting psychological distress after long-term displacement compared to stress highlights the importance of focusing on sleep health, in addition to other factors, when developing mental health-related interventions for displaced populations.

There are some limitations of the current study that should be recognized. First, the cross-sectional nature of the study makes it impossible to establish causal relationships between sleep, stress and psychological distress. Second, the study compared two distinct groups with different lengths of displacement rather than following a single group over time; therefore, the data do not capture within-group longitudinal changes. Third, while our previous research in the 1993 IDP population^11^ suggested that living conditions (lodging, financial security, safety and social life) did not significantly differ between insomniacs and good sleepers, the current study did not measure these post-displacement factors and their relative contribution to sleep and mental health. Furthermore, although bootstrapping procedures were applied to improve the robustness of the estimates, the relatively small sample size, due to challenges in recruiting hard-to-reach populations, limits the generalizability of the findings, even though observed effects ranged from medium to large. The strengths of the study should also be mentioned. The data were collected via face-to-face interviews using standardized and widely used measures. Furthermore, mental health problems were verified via a medical assessment. Finally, to the best of our knowledge, there is a lack of published studies on the relationship between stress, sleep and psychological distress among the ID population with different lengths of displacement.

## Conclusions

In conclusion, the findings of this study are in line with other studies demonstrating a high level of psychological distress in individuals with forcefully displaced backgrounds. It also extends data from a few other studies on the stability of psychological distress level over time in resettled populations. Our findings show that stress and sleep are both important correlates of psychological distress in general, with a stronger impact observed in the displaced population. Moreover, while stress might be the most important determinant of psychological distress in the early resettlement period, insomnia emerge as a more prominent predictor later, years after displacement. Although further research with larger and more representative samples is necessary, the study findings suggest that developing intervention strategies for the ID population requires a deeper understanding of how the relationship between stress, sleep, and mental health depends on the length of displacement. Addressing sleep problems, as one of the potential targets for improving the mental health and well-being of IDPs, warrants attention. In addition, interventions implemented at the early stage of displacement may prove more beneficial for preventing long-lasting psychopathologies. Given the increasing trend in refugee and ID populations worldwide, understanding the associations between sleep, stress and psychological distress in light of the length of displacement would provide valuable insights for developing effective intervention programs designed to better respond to the unique needs of forcefully displaced populations. This is especially important for countries facing prolonged conflicts, poor healthcare systems, less developed economies, and political instability.

## Data Availability

The data of this study are available from the corresponding author upon reasonable request.
